# Proteomic Investigation to Identify Anticancer Targets of *Nemopilema nomurai* Jellyfish Venom in Human Hepatocarcinoma HepG2 Cells

**DOI:** 10.3390/toxins10050194

**Published:** 2018-05-10

**Authors:** Indu Choudhary, Hyunkyoung Lee, Min Jung Pyo, Yunwi Heo, Jinho Chae, Seung Shic Yum, Changkeun Kang, Euikyung Kim

**Affiliations:** 1 College of Veterinary Medicine, Gyeongsang National University, Jinju 52828, Korea; induchoudhary2u@gmail.com (I.C.); leehy@gnu.ac.kr (H.L.); sacura8703@nate.com (M.J.P.); yunwi0510@naver.com (Y.H.); ckkang@gnu.ac.kr (C.K.); 2 Marine Environmental Research and Information Laboratory, Gunpo 15850, Korea; jinhochae@gmail.com; 3 South Sea Environmental Research Center, Korea Institute of Ocean Science and Technology (KIOST), Geoje 53201, Korea; syum@kiost.ac.kr; 4 Faculty of Marine Environmental Science, University of Science and technology (UST), Geoje 53201, Korea; 5 Institutes of Agriculture and Life Science, Gyeongsang National University, Jinju 52828, Korea; 6 Institute of Animal Medicine, Gyeongsang National University, Jinju 52828, Korea

**Keywords:** jellyfish, *Nemopilema nomurai*, HepG2 cell, venom, proteomics, MALDI/TOF/MS, 2-DE

## Abstract

*Nemopilema nomurai* is a giant jellyfish that blooms in East Asian seas. Recently, *N. nomurai* venom (NnV) was characterized from a toxicological and pharmacological point of view. A mild dose of NnV inhibits the growth of various kinds of cancer cells, mainly hepatic cancer cells. The present study aims to identify the potential therapeutic targets and mechanism of NnV in the growth inhibition of cancer cells. Human hepatocellular carcinoma (HepG2) cells were treated with NnV, and its proteome was analyzed using two-dimensional gel electrophoresis, followed by matrix-assisted laser desorption/ionization time-of-flight mass spectrometry (MALDI/TOF/MS). The quantity of twenty four proteins in NnV-treated HepG2 cells varied compared to non-treated control cells. Among them, the amounts of fourteen proteins decreased and ten proteins showed elevated levels. We also found that the amounts of several cancer biomarkers and oncoproteins, which usually increase in various types of cancer cells, decreased after NnV treatment. The representative proteins included proliferating cell nuclear antigen (PCNA), glucose-regulated protein 78 (GRP78), glucose-6-phosphate dehydrogenase (G6PD), elongation factor 1γ (EF1γ), nucleolar and spindle-associated protein (NuSAP), and activator of 90 kDa heat shock protein ATPase homolog 1 (AHSA1). Western blotting also confirmed altered levels of PCNA, GRP78, and G6PD in NnV-treated HepG2 cells. In summary, the proteomic approach explains the mode of action of NnV as an anticancer agent. Further characterization of NnV may help to unveil novel therapeutic agents in cancer treatment.

## 1. Introduction

Annual reports on public safety are raising concerns about jellyfish envenomation. Jellyfish stings are leading to a deleterious public health threat worldwide. Our ecosystem has different kinds of venomous animals, including terrestrial, such as snake, scorpion, spider, centipede, etc., and aquatic organisms, such as sea anemone, fire coral, and jellyfish [1,2]. Venomous jellyfish species are found in both cubozoans, such as *Chironex fleckeri*, and scyphozoans (*Cyanea capillata*, *Pelagia noctiluca*, and *Nemopilema nomurai*) [3,4,5,6]. We have focused on *Nemopilema nomurai* in this study; it is one of the largest jellyfish species and can grow up to 2 m in bell diameter and 200 kg in weight [7]. The giant jellyfish *N. nomurai* is endemic to the East Asian marginal seas, being principally distributed in the Bohai Sea, Yellow Sea, Northern East China Sea, and the coasts of Korea and Japan [8]. Envenomation by *N. nomurai* can result in a dermatological reaction with an intense burning sensation and erythematous eruption [9]. *N. nomurai* envenomation can also be accompanied by other types of toxicological symptoms, such as cardiotoxicity, hemolytic activity, and cytotoxic effects [6,10].

Venoms usually contain a complex mixture of hundreds of bioactive components which include small molecules, biogenic amines, peptides, and proteins [11]. Therefore, the venoms of many organisms have been investigated as a source of novel pharmacological reagents, including anticancer drugs. Current advancements in the field of proteomics and genomic research have generated a platform for the discovery of bioactive pharmaceutical components, although many researchers have evaluated the therapeutic effect of several animal venoms [12]. The existing toxicological and pharmacological research provides a broad perspective for the drug development industry, proving that these venoms and their active components can be potential sources of novel therapeutic agents.

It was previously reported that scorpion venom can inhibit the proliferation of cancer cells and primary tumors in animal models and can serve as a potential anticancer therapeutic [13,14]. Earlier research has reported that the sea anemone *Heteractis magnifica* can induce apoptosis and cell cycle arrest in lung cancer cell lines [15]. A number of studies have also demonstrated that snake venom has a strong anti-proliferative activity and antitumor effect in both in vitro and in vivo models [16,17]. Recent research has suggested that *Macrothele raveni* spider venom can induce apoptosis and inhibit the growth of leukemic K562 cells by activating caspase 3 and caspase 8 [18]. Bee venom can also effectively inhibit tumor growth. Bee venom therapy may provide beneficial effects against various types of cancer [19]. The synthetic compound Glycosphingolipid 7, which was identified in millipede *Parafontaria laminata armigera*, has an antiproliferative effect on the proliferation of melanoma (B16F10 cell lines) by suppressing the activation of the FAK-AKT and ERK1/2 pathways [20]. Moreover, a few studies have described the antitumor activity of caterpillar venom. Cecropins are set of peptides recognized in the hemolymph of the giant silkmoth (*Hyalophora cecropia*) which display anticancer activity against different types of tumor cell lines [21]. Additionally, a cardiotonic steroid (bufalin) extracted from toad venom (*Bufo gargarizans cantor*) exerts antitumor activity against numerous human cancer cell lines by inducing apoptosis and cell cycle arrest [22]. These characteristics of scorpion, snake, spider, and sea anemone venoms make them as a valuable source of therapeutic agents in cancer research.

It has been suggested that the venom of jellyfish *Chrysaora quinquecirrha* (sea nettle) has anti-tumor and antioxidant activities against Ehrlich ascites carcinoma (EAC) tumor cells [23]. Earlier, it had been demonstrated that *Nemopilema nomurai* has antioxidant activity, and that freeze-dried powder could provide potential human health benefit [24]. Jellyfishes belonging to the order Rhizostomeae are mainly used as food in Asia, especially in China and Japan, *Nemopilema nomurai* and *Rhopilema esculentum* also belongs to same order [25]. The Food and Agriculture Organization (FAO) of the United Nations has reported that jellyfish biomass can be used as a bioactive ingredient in food or medicine [25]. *N. nomurai* collagen extract can trigger the production of immunoglobulins and cytokines without causing allergic reactions, demonstrating its immune-regulatory role [26]. Jellyfish glycoprotein qniumucin can prevent articular cartilage degeneration in in vivo studies using an osteoarthritis (OA) model [27]. Our most recent report demonstrated that NnV exerts highly selective cytotoxicity in HepG2 cells via dual inhibition of the Akt and mTOR signaling pathways, but normal cells remained unaffected [28]. In the present work, we have analyzed the potential therapeutic targets of NnV at the proteomic level for the first time.

We found that the proliferation of HepG2 cells was significantly inhibited by NnV in a concentration- and time-dependent manner. In addition, we utilized two-dimensional gel electrophoresis (2-DE) coupled with matrix-assisted laser desorption/ionization time-of-flight mass spectrometry (MALDI/TOF/MS) to identify proteins with an altered abundance in HepG2 cells treated with NnV, in order to elucidate the molecular mechanisms involved in NnV anticancer therapy. The change in levels of the major proteins was also validated by Western blotting. The proteins demonstrating an altered abundance after NnV treatment may provide clues for future molecular research on the anticancer effect of NnV.

## 2. Results and Discussion

### 2.1. NnV Induces Cytotoxicity in HepG2 Cell Lines

**Figure 1:**
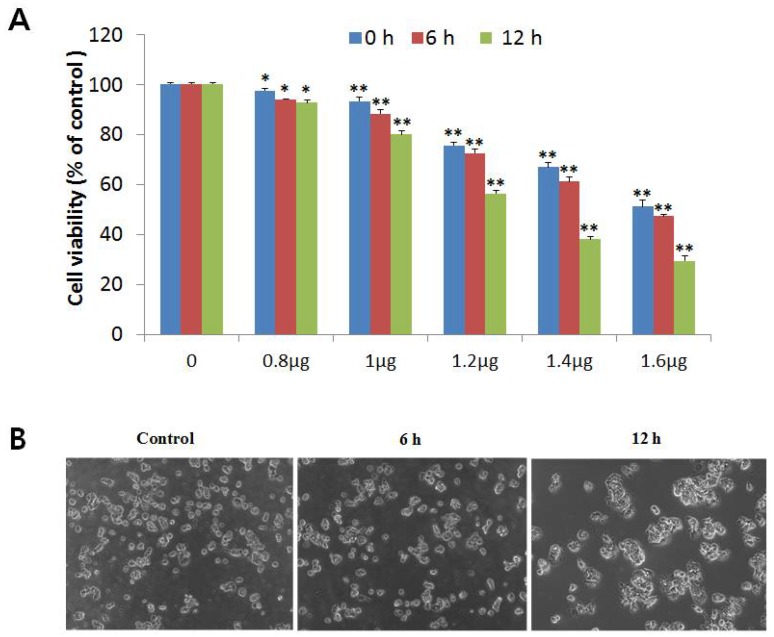
*Nemopilema nomurai* jellyfish venom (NnV) inhibits proliferation of HepG2 hepatocellular carcinoma cells. (**A**) HepG2 cells were treated with various concentrations of NnV for 6, 12, and 24 h and the cell viabilities were determined by a 3-(4,5-dimethyl-2-yl)-2,5-diphenyltetrazolium bromide (MTT) Assay; (**B**) cell morphological changes were observed using a phase contrast microscope. The left side panel is showing the untreated HepG2 cells (Control); in the middle HepG2 cells were treated with NnV at a concentration of 1.2 µg/mL for 6 h; on the right cells were treated for 12 h with same concentration. Bar graphs are the mean ± SD of the triplicate independent experiments. The * asterisk indicates a significant difference compared with control * *p* < 0.05, ** *p* < 0.01.

A MTT (3-(4,5-dimethyl-2-yl)-2,5-diphenyltetrazolium bromide) assay was used to determine the cytotoxic effect of NnV on HepG2 cells. Briefly, HepG2 cells were treated with increasing concentrations (0.8–1.6 µg/mL) of NnV for different time periods (6, 12, and 24 h). Compared with the control without NnV treatment, the viability of HepG2 cells decreased with NnV treatment in a concentration- and time-dependent manner, as shown in Figure 1A. When the cells were exposed to lower concentrations (0.8 and 1 µg/mL) of NnV there was no significant difference in cell viability at 6, 12, or 24 h post-treatment. After 24 h of incubation with NnV at concentrations of 1.2, 1.4, and 1.6 µg/mL, cell proliferation decreased to 56.3%, 38.1%, and 29.4%, respectively. Treatment with 1.2 µg/mL of NnV for 24 h resulted in a significant decrease in cell viability compared with that of the control without NnV treatment. The IC50 value of NnV concentration needed for 50% inhibition of cell proliferation was determined to be 1.2 µg/mL using probit analysis. This concentration was used for further experiments. For proteomic analysis, we treated HepG2 cells with 1.2 µg/mL (IC50 value) of NnV. The phase contrast microscopic analysis was performed to examine the morphological characteristics of HepG2 cells (Figure 1B). In comparison to the control, NnV treated HepG2 cells showed cell shrinkage, loss of adhesion with round shape, necrosis, and formation of apoptotic fragments. On the other hand, HepG2 control cells grew properly and had normal growth. Our results demonstrate that NnV significantly decreased the number of viable cells in a concentration- and time-dependent manner and had antiproliferative activity.

### 2.2. 2D PAGE Analysis of Untreated and NnV Treated H9c2 Cells

**Figure 2:**
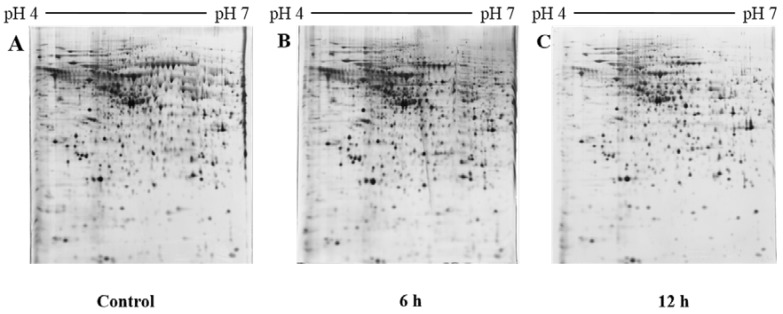
Comparison of patterns shown by two-dimensional gel electrophoresis (2-DE) images between HepG2 cells. (**A**) Control; (**B**) treated with NnV at a concentration of 1.2 µg/mL for 6 h and (**C**) 12 h. For the first dimension, 300 µg of total protein was resolved on 18 cm IPG dry strips (pH 4–7 L), and the second dimension was carried out using 12% SDS-PAGE gels. 2-DE gels were silver stained and scanned by an Epson perfection V 700 photo scanner. Three independent replicate gels were run for further statistical analysis.

**Figure 3:**
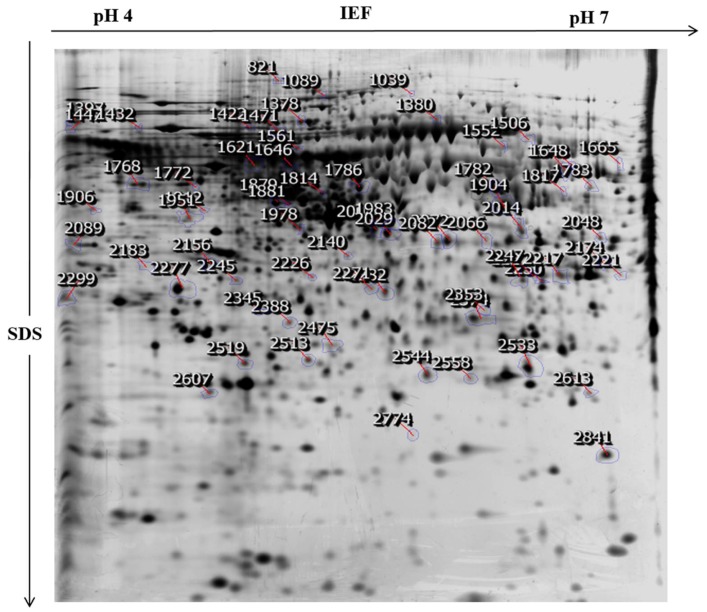
Image of 2-DE proteomic analysis of NnV treated HepG2 cells. The representative 2D image was generated using Progenesis Same Spots software, and protein spots were further analyzed by matrix-assisted laser desorption/ionization time-of-flight mass spectrometry (MALDI/TOF/MS). The position of differentially expressed proteins was assigned with blue boundaries and red arrows. Protein spots with numbers were hand-picked to perform in-gel digestion.

To investigate in depth molecular changes at protein level after NnV treatment we performed comparative proteomic analysis to determine protein level changes between HepG2 cells treated with or without NnV at IC50 for 6 or 12 h. To determine which proteins had altered abundance in HepG2 cells after NnV treatment proteins from the control and NnV treated HepG2 cells (6 h and 12 h) were examined by 2-DE. Three biological replicates of the experiment were performed to obtain the reproducibility of the gels. Approximately 1000 protein spots were visualized after silver staining. All gels were run in triplicates. The patterns of 2-DE spots present in all gels from the three independent experiments showed no significant differences, corroborating the reproducibility of our experiment. In Figure 2, representative 2-DE images of control and NnV treated (6 and 12 h) protein samples of HepG2 cells are shown. All 2-DE gel images were scanned and analyzed using Progenesis Same Spots software (Nonlinear Dynamics, New Castle, UK). After performing automatic spot detection and image analysis, 70 protein spots with a ≥1.5 fold change in intensity were found with differential abundances and statistical significance (ANOVA *p*-value ≤ 0.05). Representative fused 2-DE gel images of the control and NnV treated samples (6 h and 12 h) are shown in Figure 3. These 70 differentially expressed protein spots were subjected to MALDI/TOF/MS analysis.

### 2.3. Identification of the Differential Abundant Proteins by MALDI/TOF/MS and Western Blotting

**Figure 4:**
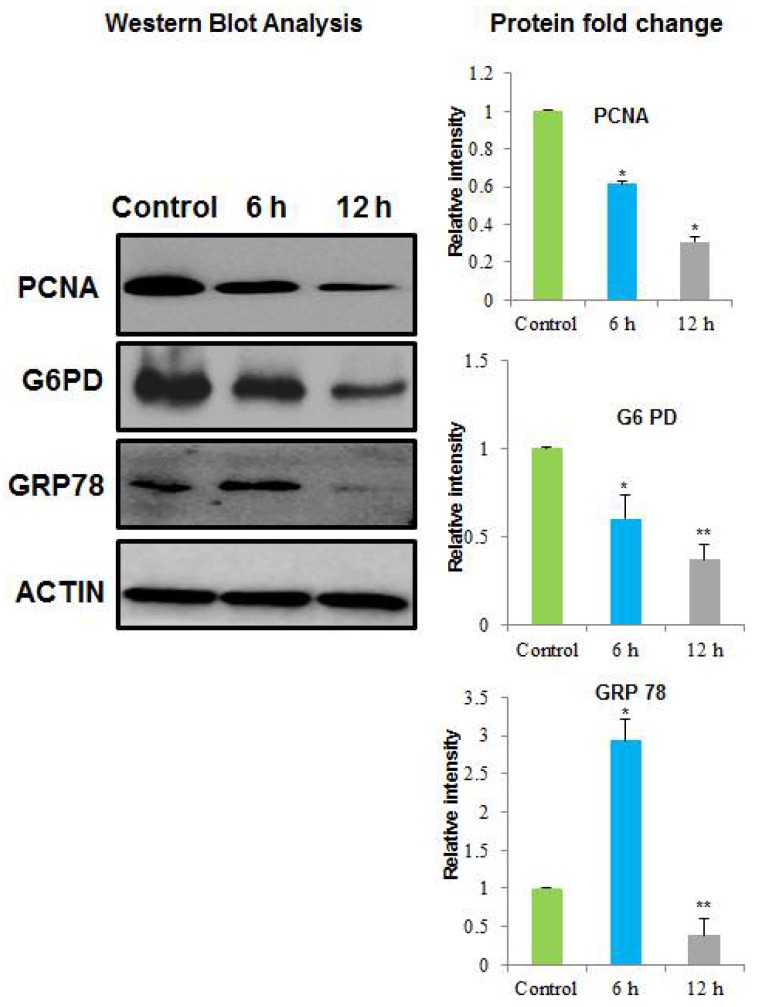
Western blot analyses for confirming the altered protein volumes as identified by MALDI/TOF/MS. Western blots show representative protein bands for GRP78, PCNA, and G6PD; β-actin was used as the loading control. The densitometric analyses of Western blots were performed by Image Lab software. The bar graph represents the mean ± SD of the amount of each protein after NnV treatment in comparison to control. The * asterisk indicates significant differences when compared with the control * *p* < 0.05, ** *p* < 0.01.

The 70 differentially expressed proteins were excised manually from the 2-DE gels for MALDI/TOF/MS analysis. Of the 70 protein spots, only 24 proteins were successfully identified by MALDI/TOF/MS. Results are shown in Table 1. Of the 24 proteins identified by MALDI/TOF/MS, the amounts of 14 proteins reduced, while 10 proteins showed an increased abundance with NnV treatment. The relative spot intensity of these differentially expressed proteins, as well as their fold change and P values, were summarized in the Supplementary Materials Tables S1 and S2, respectively. One of the 14 proteins with decreased abundance was PCNA. PCNA plays a pivotal role in numerous cellular processes, for instance DNA replication, cell cycle progression, transcription, and chromosome segregation [29,30,31]. Consequently, it has been previously reported that the expression level of PCNA was higher in cancer cells and cancer tissues, such as breast, gastric, and lung cancer [32,33,34,35]. It has been found that novel classes of the compound known as PCNA inhibitor (PCNA-I1) can directly bind to PCNA, stabilize PCNA trimers, decrease PCNA–chromatin association, and inhibit tumor cell growth by inducing a cell cycle arrest [36]. Overexpression of PCNA is a remarkable biomarker for tumor diagnosis [32,33]. Thus, PCNA might be a possible target for cancer therapy. In the present study, the protein level of PCNA significantly decreased in response to NnV treatment, which was further confirmed by Western blot analysis (Figure 4). Hence, the reduced amount of PCNA in NnV treated HepG2 cells might contribute to the antiproliferative and anticancer effect of NnV.

Another protein with a reduced amount, identified by proteomic analysis in this study, was glucose-regulated protein 78 (GRP78). GRP78 belongs to the heat shock protein 70 (HSP70) family. It is best known as a immunoglobulin heavy chain binding protein (BiP) [37]. GRP78 is mainly located in the lumen of the endoplasmic reticulum and acts as a molecular chaperone. It also resides in the plasma membrane, cytoplasm, mitochondria, and nucleus. Furthermore, it is present in the cellular secretions of tumor cells [38]. GRP78 is involved in protein folding and assembly, degradation of the targeted misfolded protein, ER Ca^2+^ binding, and the regulation of the activation of transmembrane ER-stress sensors [38,39]. It also has antiapoptotic properties [39,40]. GRP78 is also involved in tumor cell proliferation, apoptosis resistance, tumor metastasis, and angiogenesis [41]. Hence, it plays a vital role in cancer development. GRP78 overexpression can result in opposition to a wide range of chemotherapeutic agents in the different classes of tumor, including lung, bladder, gastric, breast stomach, and epidermoid carcinoma [42,43,44,45,46]. It has been reported that knockdown of GRP78 can inhibit tumor cell proliferation and suppress metastasis and growth in xenograft models [47]. Our 2 DE results revealed that amount of GRP78 was significantly reduced in NnV treated HepG2 cells; this was confirmed by Western Blot analysis (Figure 4). Therefore, a reduced level of GRP78 in NnV treated HepG2 cells might have induced apoptosis and conferred the anticancer effect of NnV.

Glucose-6-phosphate dehydrogenase (G6PD), a central enzyme in the pentose phosphate pathway was also identified in the present study. It can generate NADPH and ribose-5-phosphate in rapidly growing cells, which is necessary for the synthesis of RNA and DNA [48]. The generated NADPH serves as a vital component for glycolysis and is involved in various reductive biosynthetic reactions. Glycolysis maintains the levels of glutathione required to carry out the detoxification of free radicals and peroxides [49,50]. Consequently, G6PD might be able to induce cancer cell survival and growth through the pentose phosphate pathway by creating NADPH and ribose [51,52,53]. Moreover, G6PD expression is highly elevated in numerous types of cancers such as breast, colon, leukemia, melanoma, cervical, prostatic, and endometrial cancers [54,55,56]. Previous studies have shown that ectopic expression of G6PD in anchorage-independent NIH 3T3 cells resulted in higher levels of NADPH and glutathione which promoted cell growth [57]. Moreover, in nude mice, G6PD gene transformed NIH 3T3 cells showed tumorigenic properties, indicating that G6PD may act as a possible oncogene [58]. The up-regulated level of G6PD in various categories of cancer cells suggests that G6PD might have potential future applications as a diagnostic and prognostic marker in cancer therapy. A recent report has demonstrated that after RNA interference and down-regulation of G6PD in the IFN signaling pathway HBV replication is reduced five-fold, while overexpression of G6PD resulted in the amendment of the IFN signaling pathway [59]. Recent research has revealed that dehydroepiandrosterone (DHEA) and shRNA technologies can inhibit G6PD activity, increase apoptosis, and decrease the proliferation and migration of Hela cells [60]. Buthionine sulfoximine (BSO) functions as a glutathione depletion agent, it can reduce colony formation of G6PD-overexpressing cells in soft agar [60], suggesting that G6PD inhibitors can be used potentially to repress the cancer cell growth. Therefore, G6PD might be considered as an excellent target for cancer therapy. In the current study, 2DE and MALDI/TOF/MS results showed that the protein level of G6PD was significantly reduced in HepG2 cells treated with NnV for 6 or 12h compared with that in control.

The reduced abundance of G6PD was validated by Western blotting analysis (Figure 4), clearly indicating that NnV could inhibit the proliferation of HepG2 cells. Therefore, G6PD may contribute to the therapeutic function of NnV in cancer therapy. The present results revealed that the expression level of elongation factor 1γ (EF1γ) was decreased after NnV treatment. EF1γ is a well-known subunit of elongation factor-1 (EF-1) and has a crucial role in protein synthesis, it mediates the transport of aminoacyl tRNA to 80S ribosomes, resulting in the elongation of polypeptide chains [61]. EF-1γ is a substrate of the cdc2 kinase, a maturation-promoting factor that monitors entry into the M-phase of the cell cycle in all eukaryotic cells [61,62]. Previous studies have shown that EF-1γ mRNA is overexpressed in breast, colorectal, gastric, hepatocellular, pancreatic, and esophageal carcinoma [62,63,64,65,66,67]. Earlier clinicopathologic studies have revealed that EF-1γ mRNA is overexpressed in 15 of the 22 gastric carcinoma cases that tested positive for vascular permeation [64]. Moreover, 9 of 10 patients showed severe vascular permeation in histologic slides with a 10-fold higher overexpression of EFI-γ mRNA [64]. Therefore, there is a remarkable correlation between the overexpression of EFI-γ mRNA and vascular permeation, suggesting that the overexpression of EFI-γ may be a valuable diagnostic marker for gastric carcinomas. In our experiment, the level of EFI-γ protein in NnV treated HepG2 cells was reduced, suggesting its putative role in the action of NnV as an anticancer agent. Dynamin-related protein 1 (Drp1), a member of the dynamin family of GTPases, had a reduced amount in NnV-treated HepG2 cells. Drp1 is needed for mitochondrial fission. Drp1 expression has been reported to be up-regulated in distinct types of cancers, including breast and lung cancers [68]. The siRNA-mediated DRP1 knockdown can result in the gathering of elongated mitochondria in colon cancer cells [69]. In addition, DRP1 down-regulation can decrease the level of cell proliferation and intensify colon cancer apoptosis [69]. It has been reported that DRP1 inhibition can lesson cancer cell growth and escalate apoptosis in both in vivo and in vitro models of various cancers, including colon, breast, lung, and cervical cancers [69,70,71,72]. These findings suggest that DRP-1 can be utilized as a therapeutic target for various kinds of cancers. Our results also showed that DRP-1 protein abundance decreased in NnV treated HepG2 cells compared with that in the control, further supporting the function of NnV as an anticancer agent. Another protein with reduced abundance level, Nucleolar and spindle-associated protein (NuSAP), a novel microtubule-associated protein, was found in this study. NuSAP is expressed in swiftly proliferating cells, and is an essential regulator of mitosis [73]. NuSAP is involved in various biological processes, including the organization of mitotic spindle assembly, chromosome segregation, sister chromatid segregation, and cytokinesis [73,74]. Numerous research groups have suggested that NuSAP is associated with cancer [74]. NuSAP is elevated in hepatocellular carcinomas. Therefore, it has been used as an excellent prognostic factor for hepatic carcinoma [75]. Furthermore, NuSAP has been recognized as a malignancy-risk gene associated with cancer progression in breast cancer patients [76]. Previous reports have shown that the NuSAP gene is highly expressed in aggressive melanomas at the mRNA level compared to its expression in less aggressive cells [77]. It has been found that NuSAP is down-regulated about 2.5-fold in response to methionine stress in CAPAN1 pancreatic adenocarcinoma cells [78]. In a recent study, in vivo knockdown of NuSAP by antisense oligonucleotide morpholino technology altered the migration of neural crest cells in zebrafish embryos [79]. Therefore, diminution of NuSAP expression resulted in the dwindling of cell migration, whereas, NuSAP overexpression encouraged cell migration in zebrafish embryos [79]. These results have demonstrated that NuSAP is associated with apoptosis and cell migration, resulting in carcinogenesis. A recent study has reported that NuSAP expression is up-regulated in oral squamous cell carcinoma (OSCC) at both protein and mRNA levels [80]. NuSAP knockdown resulted in the suppression of cellular proliferation and intensified the anti-tumor activity of paclitaxel by initiating apoptotic pathways [80]. This suggests that NuSAP is an essential biomarker for OSCC, and that NuSAP might serve as an active therapeutic target using NnV.

Activator of 90 kDa heat shock protein ATPase homolog 1 (AHSA1) is known as a chaperone of heat shock 90 kDa (HSP90). It is essential for the stimulation of the ATPase activity of HSP90 [81]. It has also been suggested by other investigators that AHSA1 is up-regulated in osteosarcoma (OS). The silencing of AHSA1 can inhibit cell growth, increase cell apoptosis, and decreased migration and invasion of MG-63 and Saos2 cells [82]. Furthermore, AHSA1 silencing in OS can improve the activity of negative regulators of the Wnt/β-catenin signaling pathway, induce cell apoptosis, and reduce cell proliferation [81,82]. AHSA1 may perform an oncological role in OS. It provides an opportunity to establish a potential therapeutic target in OS. In our current work, treatment with NnV decreased the protein level of AHSA1 in HepG2 cells, further supporting the anticancer activity of NnV.

The adrenal cortex Steroid 21-hydroxylase gene (CYP21A2) catalyzes 17-hydroxyprogesterone (17-OHP) into 11-deoxycortisol (11-DOF) and progesterone into 11-deoxycorticosterone (11-DOC). These steroids are converted into cortisol and aldosterone, respectively. Congenital adrenal hyperplasia (CAH) disorder is caused due to deficiency in the 21-hydroxylase gene (CYP21A2). CAH is an autosomal recessive disorder which can act as an important factor in the development of adrenocortical tumors. The transcription factor p53 is a significant tumor suppressor that acts as a hemostatic gene, organizing diverse cellular processes. It has been reported that p53 activation in several types of hepatic cells leads to the increased secretion and expression of the 21-hydroxylase gene (CYP21A2). CYP21A2 was identified as a p53 target gene that demonstrates a novel non-cell-autonomous tumor-suppressive regulation mediated by p53, which can maintain organism homeostasis and prevent malignant disease. Interestingly, Steroid 21-hydroxylase (CYP21A2) protein level was incereased after NnV treatment for 6 or 12 h in comparison with the control, which hints towards NnV dependent p53 pathway modulation.

### 2.4. Ontological Classification of Differentially Abundant Proteins

**Figure 5:**
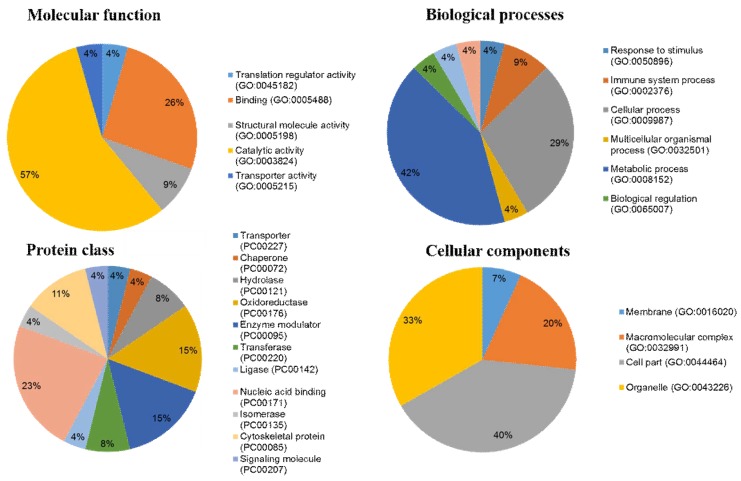
Gene ontology (GO) enrichment analyses of proteins with altered amounts. The identified proteins were categorized into four groups; Molecular function, Biological process, Protein class, and Cellular components, using the Panther classification system (http:www.pantherdb.org/).

According to gene ontology classification, the 24 identified proteins with altered amounts in NnV treated HepG2 cells were classified on the basis of molecular function, biological process, protein class, and cellular components. Based on molecular component ontology, the two major functional categories of these proteins were catalytic activity (57%) and binding (26%). Some proteins exhibited structural molecule activity (9%) and translation regulator or transporter activity (4%). In the biological process group, most proteins were associated with the metabolic process (42%), cellular process (29%), immune system process (9%), response to stimulus, biological regulation, cellular component organization or biogenesis, localization, metabolic process, and multicellular organismal process (4%). The most important categories of proteins were a response to stimulus (4%) and immune system process (9%), indicating the nature of NnV. Further these proteins were divided on the basis of functional protein classes; the most abundant class is of nucleic acid binding proteins (23%). Many proteins belonged to the following protein classes: oxidoreductase and enzyme modulator (15%), cytoskeletal protein (11%), hydrolase (8%), transferase (8%), chaperone, transporter, signaling molecule, kinase, isomerase, and ligase (4%). In terms of cellular components, the majority of these proteins were localized to the cell part (40%), organelle (33%), and macromolecular complex (20%). Only a few proteins were confined to the cell membrane (7%). Among these annotations, the most prevailing terms in the molecular function, biological process, protein class, and cellular components were the catalytic activity (57%), metabolic process (42%), oxidoreductase and enzyme modulator (15%), and cell part (40%) respectively (Figure 5).

**Figure 6:**
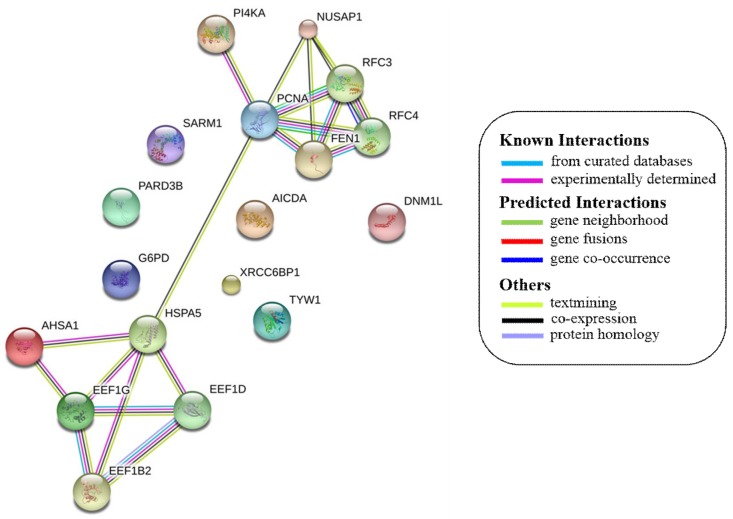
Interaction network of identified differentially expressed proteins using STRING database V 10.5 (http://string-db.org). String pathway analysis of the proteins affected by NnV treatment in HepG2 cells. The types of interactions are represented by different colored lines as shown on the right panel.

Most of the above differentially expressed proteins are involved in cancer regulation. To predict protein–protein interactions and protein complexes, along with putative pathways, the above proteins were subjected to STRING (Search Tool for the Retrieval of Interacting Genes/Proteins) analysis. Generally, proteins were sorted into two different groups, as shown in Figure 6. Both the groups were connected to each other. The first group consisted of PCNA1 and NuSAP1, which are well-known cancer markers, as mentioned above. PCNA1 seems to be in the center of the group and shows interactions with many other proteins involved in the cancer pathway, such as RFC3 and RFC4, which are well-known prognostic cancer markers [83,84,85]. Another group consists of EEF1G and AHSA1 (Activator of 90 kDa heat shock protein ATPase homolog 1), which were overexpressed in several types of carcinomas and may act as valuable prognostic cancer markers. EEF1G and AHSA1 also appeared to show a tight cluster interaction network with other proteins such as HSPAS and EEF1D. Both HSPA5 (heat shock 70 kDa protein 5) and EEF1D were reported to be expressed at an elevated level in different types of cancers and are associated with tumorigenicity. Also they act as a biological marker for cancer grade, metastases, and prognosis [86,87,88]. The string interaction and KEGG (Kyoto Encyclopedia of Genes and Genomes) pathway of important cancer marker proteins are shown in Table 2.

To the best of our knowledge, this is the first report showing that NnV can induce apoptosis of cancer cells at the proteome level. NnV treatment resulted in diminished levels of proteins involved in DNA replication, cell cycle progression, transcription, tumor cell proliferation, apoptosis resistance, tumor metastasis, and angiogenesis. A few proteins have also been dubbed as oncoproteins due to their overexpression in different types of cancer. Further study on these targets may provide a platform for understanding the molecular mechanisms involved in NnV induced apoptosis in HepG2 cells. In the near future, more comprehensive efforts need be carried out to understand the detailed mechanisms of NnV as a cancer therapeutic agent.

## 3. Materials and Methods

### 3.1. Sample Collection and Preparation

The samples of *N. nomurai* jellyfish were harvested from the sea surrounding Gunsan city, South Korea. Tentacles were dichotomized and moved immediately in ice to the laboratory for further research. Nematocysts were collected using the previously used method with a slight modification [89]. The dissected tentacles were washed with cold sea water to separate debris, after that 3 volumes of cold sea water was added and kept for overnight shaking at 4 °C. The tentacles free sea water extract was centrifuged at 1000× *g* for 5 min, the pellet was collected, and then washed three times with sea water. Sedimented tentacles were further autolyzed overnight in fresh sea water at 4 °C, and the autolysis process was reiterated for 3–4 days. Lastly, the settled nematocysts were collected and washed numerous times with fresh sea water. Nematocysts were collected at 500× *g* for 5 min. Further pellets (nematocysts) were lyophilized and stored at −20 °C until further use.

### 3.2. Venom Extraction and Preparation

Freeze-dried nematocysts were used to isolate the venom using a previously reported method [90], with a minor amendment. Next, venom was sequestered from 50 mg of nematocyst using glass beads (approximately 8000 beads; 0.5 mm in diameter) and 1 mL of ice-cold phosphate buffer saline (PBS, pH 7.4). The above samples were vortexed for 30 s, repeated 5 times with intermittent cooling on ice. The venom extracts were then transferred to new Eppendorf tubes and centrifuged (22,000× *g*) at 4 °C for 30 min. In the present study, the supernatant was used as jellyfish venom. The Bradford technique [91] was used to quantify the protein concentration of the venom.

### 3.3. Cell Culture

HepG2 cells were bought from the American Type Culture Collection (ATCC, Manassas, VA, USA) and were maintained in Dulbecco’s Modified Eagle Medium (DMEM), supplemented with 10% heat-inactivated fetal bovine serum (FBS), 100 µg/mL Penicillin-Streptomycin-Amphotericin B solution at 37 °C, in a humidified atmosphere with 5% CO_2_. Every 2–3 days the medium was changed, the cell culturing was done until 70% confluence was attained. The above cells were treated with 1.2 µg/mL NnV for 6 h and 12 h.

### 3.4. MTT Assay for Cell Viability

The cell viability was checked by MTT assay to measure the cytotoxicity of NnV in HepG2 cells. HepG2 cells were seeded at a density of 4 × 10^4^ cells/well in 24-well plates for 24 h. HepG2 cells were treated with different concentrations of NnV for 24 h. After 24 h, 50 µL of MTT dye (5 mg/mL) was added to each well and the cells were further incubated for 3 h at 37 °C. 100 µL of dimethyl sulfoxide (DMSO) was added to each well after removing the medium. To solubilize the generated formazan salts the 24-well plate was kept on a shaker for 10 min. The optical density at 540 nm was assessed using a GENios microplate spectrophotometer (PowerWave™XS, BioTek Instruments, Inc., Winooski, VT, USA) and was measured to determine the cell viability. The IC_50_ was determined by Probit analysis [92]. The morphological changes in HepG2 cells were examined after NnV treatment under the phase contrast microscope. HepG2 cells without any treatment were used as the control. MTT assays were performed in triplicates for each dosage and time period.

### 3.5. Protein Extraction and Sample Preparation

For proteome analysis, the human liver cancer cell line HepG2 was seeded in tissue culture plates at 5 × 10^4^ cells/mL and incubated overnight, then treated with NnV for 6 and 12 h. Untreated cells were used as the control sample. At the end of the treatment the cells were washed with ice-cold 1× phosphate-buffered saline and then homogenized in 2D lysis buffer (7 M Urea, 2 M thiourea, 4% CHAPS (3-[(3-cholamidopropyl) dimethylammonio]-1 propanesulfonate, 10% DTT, 0.5% IPG buffer, and 1% proteinase inhibitor) on ice. Samples were vortexed for 30 min at 4 °C and cellular debris were removed by centrifugation (14,000 rpm for 15 min at 4°C). Proteins were precipitated by adding equal volumes of 20% TCA to cell lysis supernatant and incubating for 30 min on ice. Protein precipitates were centrifuged at 14,000 rpm for 15 min and the supernatant was discarded. TCA precipitated proteins were washed thrice with ice-cold acetone and centrifuged as above. After that, pellets were vacuum dried for 10–15 min. Subsequently, dried pellets were resuspended in 200–300 µL of sample buffer (7 M urea, 2 M thiourea, and 4% CHAPS). The sample was determined for protein concentration by Bradford assay and stored at −80°C until further use.

### 3.6. Two-Dimensional Gel Electrophoresis and Image Analysis

For 2D gel electrophoresis, 300 µg of protein samples were diluted to 340 µL by mixing of the 2D sample buffer (7 M urea, 2 M thiourea, 4% CHAPS, 10 mg/mL DTT, 1% ampholytes, and a few grains of bromophenol blue). These protein samples were applied to the immobiline 18 cm TM Dry strip (pH 4–7), followed by rehydration overnight at room temperature. Isoelectric focusing was conducted using the Ettan IPGphor system (GE Healthcare, Salt Lake City, UT, USA) with the following conditions: 50 V for 1:00 h, 200 V for 1:00 h, 500 V for 0:30 h, gradient 4000 V for 0:30 h, 4000 V for 1:00 h, gradient 10,000 V for 1:00 h, 10,000 V for 13:00 h, and 50 V 3:00 h at 20 °C. After Isoelectric focusing, the gel strips were firstly reduced with equilibration buffer (50 mM Tris-HCl (pH 8.8), 6 M urea, 30% glycerol, 2% SDS, and 0.01% bromophenol blue containing 1% *w*/*v* DTT for 15 min. Again, the gel strips were washed with the same buffer (50 mM Tris-HCl (pH 8.8), 6 M urea, 30% glycerol, 2% SDS, and 0.01% bromophenol blue, replacing the DTT with 2.5% *w*/*v* iodoacetamide) for 15 min. Equilibrated strips were placed over 12% SDS polyacrylamide gels (18 cm × 20 cm × 1.5 mm) and then covered with 0.5% (*w*/*v*) agarose made in the SDS electrophoresis running buffer containing a minute amount of bromophenol blue. Electrophoresis was performed in a PROTEAN II xi cell gel electrophoresis unit (Bio-Rad, Hercules, CA, USA) with a constant current of 20 mA/gel at 20 °C until the dye front reached the end of the gels. The electrophoresis 2-DE gels were then silver stained, as similar to the method reported by [93]. The 2D gel images were achieved by a Epson perfection V 700 photo scanner (Epson, Suwa, OU, Japan), and image analysis was carried out using Progenesis Same Spots software (Nonlinear Dynamics, Newcastle, UK).

### 3.7. In-Gel Digestion

The protein spots which show differential expression were manually excised from the preparative gels to perform in-gel digestion. In-gel digestion of the proteins was performed according to the method previously described, with some modifications [94]. The gel pieces were washed with destaining solution containing 30 mM potassium ferricyanide and 100 mM Na_2_S_2_O_3_ (50%/50% *v*/*v*) for 10 min. For the dehydration step, the gel pieces were rinsed in 100% acetonitrile for 10 min and dried using lyophilizer equipment. For the reduction step, 50 µL of reduction solution (10 mM DTT in 100 mM ammonium bicarbonate) was added to the gel pieces, which were incubated for 45 min at 56 °C. After removing the reduction solution the alkylation solution (100 mM iodoacetamide in 100 mM ammonium bicarbonate) was added to the gel pieces for 45 min in the dark, at room temperature. The alkylation solution was removed, and the gel pieces were incubated in trypsin (Promega, Madison, WI, USA) at a final concentration of 2 ng/µL in 10 µL of 50 mM NH_4_HCO_3_ and incubated on ice for 45 min. Next, the trypsin was removed, and the proper amount of 50 mM NH_4_HCO_3_ was added to the gel pieces; digestion was carried out overnight at 37 °C. In the extraction step, the tryptic–peptide mixture was pooled with extraction buffer containing 100% acetonitrile and 50% trifluoroacetic acid and then lyophilized in a speed vacuum.

### 3.8. MALDI/TOF/MS Analysis and Database Searching

One microliter of HCCA matrix solution (α-acyano-4-hydroxycinnamic acid) and 1 µL of extraction buffer were added to the lyophilized peptide and spotted onto a recently washed MALDI-TOF target plate. Next, the samples were completely crystallized by air drying for 10 min at room temperature. The samples were analyzed on a Voyager-DE STR mass spectrometer (Applied Biosystems, Franklin Lakes, NJ, USA) using reflection/delayed extraction mode. The mass spectra were acquired over a mass range of 800–3000 Da. To carry out protein identification, the peptide mass fingerprinting data were searched against the SwissProt database using Mascot server (Matrix science http://www.matrixscience.com). The following factors were considered throughout the search: taxonomy as Homo sapiens, tryptic digest with one missed cleavage is allowed, peptide mass tolerance of 100 ppm for the fragment ions, fixed modification as the alkylation of the carbamidomethylation, and oxidation of methionine as a variable modification. A protein scores greater than 56 were considered statistically significant (*p* < 0.05).

### 3.9. Western Blotting

HepG2 cells were treated with NnV for 6 h, 12 h and untreated cells were used as control samples. After treatment, the cells were rinsed thrice with ice-cold phosphate buffered solution (PBS) and the whole cellular protein was lysed on ice with radioimmunoprecipitation assay buffer (RIPA; iNTRON Biotechnology, Seongnam, Gyeonggi-do, Korea) containing a protease inhibitor cocktail. Next, the cell lysates were centrifuged at 22,000× *g* for 30 min at 4 °C, cellular debris were removed, and the supernatant was collected. Protein concentrations of the extracted samples were determined via Bradford assay. Equivalent amounts of protein samples (10 µg) were subjected to a 10% SDS-PAGE at 100 V and then were transferred to a PVDF membrane (Bio-Rad Laboratories, Hercules, CA, USA) at 25 V for 30 min. The membranes were blocked with 5% non-fat milk in Tris-buffered saline and 0.1% Tween 20 for 2 h at room temperature. Thereafter, the membranes were incubated with the following respective primary antibodies: GRP 78 (N-20): sc-1050 (1:500, Santa Cruz Biotechnology, Inc., Dallas, TX, USA), PCNA (PC10): sc-56 (1:500, Santa Cruz Biotechnology, Inc.), and Anti-Glucose 6 phosphate Dehydrogenase antibody ab76598 (1:500, Abcam, Cambridge, UK) incubated overnight at 4 °C temperature. Membranes were also probed with an anti β-actin (1:2000 Millipore) primary antibody as a loading control. After that, the membranes were washed thrice with TBST buffer for 10 min and were incubated with the appropriate secondary antibodies (1:2000 dilutions) for 1 h at RT. Target proteins were developed by enhanced chemiluminescence (ECL, Amersham Biosciences, Buckinghamshire, UK) and scanned using ChemiDoc™ XRS (Bio-Rad, Hercules, CA, USA). Further quantitative examination was completed with Image Lab™ software (Bio-Rad, Hercules, CA, USA).

### 3.10. Statistical and Bioinformatics Analysis of Protein Identified by MALDI/TOF/MS

For the statistical analysis of differentially expressed proteins, One Way Analysis of Variance (ANOVA) was used to evaluate the significance of the difference between the two mean values. *p* < 0.05 and *p* < 0.01 were considered to be statistically significant. The proteomic data were analyzed using the Panther classification system (http:www.pantherdb.org/) to classify data in terms of molecular function, biological process, protein class, and cellular components [95]. The interaction network of the differentially expressed proteins was performed using the online STRING database V 10.5 (http://string-db.org) [96].

## Figures and Tables

**Table 1:** Identification of differentially expressed proteins using MALDI/TOF/MS from HepG2 cells treated with *N. nomurai* venom.

Spot No ^1^	Accession Number ^2^	Protein Name	Theoretical MW/Pi ^3^	Mascot Score ^4^	Coverage % ^5^	Matched Peptides ^6^	Biological Features
**Down-regulated Proteins**							
**2277**	PCNA_HUMAN	Proliferating cell nuclear antigen	29,092/4.57	144	72%	16	DNA repair, DNA Regulation, and mismatch repair.
**1648**	KIF28_HUMAN	Kinesin like protein KIF 28P	109,098/8.68	58	9%	8	Mitochondrian organization and organelle transport along microtubule.
**2299**	GRP78_HUMAN	78 kDa glucose -regulated protein	72,402/5.07	69	17%	9	Negative regulation of the apoptotic process and negative regulation of transforming growth factor beta receptor signaling pathway.
**2008**	AHSA1_HUMAN	Activator of 90 kDa heat shock protein ATPase homolog 1	38,421/5.41	72	28%	9	Positive regulation of ATPase activity.
**1768**	NUSAP_HUMAN	Nucleolar and spindle-associated protein 1	49,593/9.92	61	32%	12	Establishment of mitotic spindle localization, mitotic chromosome condensation, and positive regulation of mitosis.
**2245**	NDUF7_HUMAN	NADH dehydrogenase [ubiquinone] complex 1, assembly factor 7	49,435/8.47	68	11%	5	Methylation, mitochondrial respiratory complex 1 assembly, and methyltransferase activity.
**1978**	DNM1L_HUMAN	Dynamin-1-like protein	82,339/6.37	57	14%	6	Positive regulation of the apoptotic process and positive regulation of intrinsic apoptotic signaling pathway.
**2183**	ATLA3_HUMAN	Atlastin-3	60,960/5.43	58	10%	6	GTP catabolic process, golgi organization, endoplasmic reticulum organization and, homooligomerization.
**1772**	HNRH1_HUMAN	Heterogeneous nuclear ribonucleoprotein H	49,484/5.89	74	33%	10	RNA Processing, regulation of RNA splicing, POLY(A) RNA binding, and poly(U) RNA binding.
**1817**	EF1G_HUMAN	Elongation factor 1-gamma	50,429/6.25	58	15%	6	Translational elongation, cellular protein metabolic process, translation, and gene expression.
**Up-regulated Proteins**							
**1665**	G6PD_HUMAN	Glucose-6-phosphate 1-dehydrogenase	59,675/6.39	82	16%	8	Pentose-phosphate shunt, oxidative branch, Glucose 6-phosphate metabolic process, NADP metabolic process, and NADPH (nicotinamide adenine dinucleotide phosphate-oxidase) regeneration.
**2245**	ATP23_HUMAN	Mitochondrial inner membrane protease ATP23 homolog	28,690/8.30	57	29%	4	Double strand break via non homologous end joining and protein phosphorylation.
**2089**	DNM3A_HUMAN	DNA (Cytosine-5)-methyltransferase 3A	103,390/6.19	58	18%	16	DNA methylation, *S*-adenosylhomocysteine metabolic process, regulation of transcription from RNA poly II promoter, and regulation of gene expression by genetic imprinting.
**1648**	TYW1_HUMAN	*S*-adenosyl-l-methionine-dependent tRNA 4-demethylwyosine synthase	84,732/6.42	57	7%	7	tRNA processing.
**1646**	CP21A_HUMAN	Steroid 21-hydroxylase	56,251/7.71	57	11%	5	Glucocorticoid biosynthetic process and steroid metabolic process.
**2353**	AICDA_HUMAN	Single-stranded DNA cytosine deaminase	24,337/9.50	68	47%	9	mRNA processing, DNA demethylation, cell differentiation, and protein binding.
**1906**	UBP15_HUMAN	Ubiquitin carboxyl-terminal hydrolase 15	113,602/5.06	56	18%	10	Transforming growth factor beta receptor signaling pathway, BMP signaling pathway, and protein deubiquitination.
**2029**	PI4KA_HUMAN	Phosphatidylinositol 4-kinase alpha	233,622/6.43	70	28%	62	Signal transduction, phosphatidylinositol-mediated signaling, and phospholipid metabolic process.
**2014**	SYHC_HUMAN	Histidine-tRNA ligase	57,944/5.72	64	34%	17	Histidyl-tRNA aminoacylation, cellular metabolism, tRNA amino acylation for protein translation, and protein biosynthesis.
**821**	DHB13_HUMAN	17-beta-hydroxysteroid dehydrogenase 13	33,976/9.14	65	46%	10	Oxidoreductase activity.
**2841**	SARM1_HUMAN	Sterile alpha and TIR motif-containing protein 1	80,365/6.14	57	20%	9	Toll like receptor signaling pathway, regulation of dendrite morphogenesis, and regulation of neuron death.
**2282**	ZSC31_HUMAN	Zinc finger and SCAN domain-containing protein 31	48,233/6.42	63	14%	5	Transcription and transcription regulation.
**2217**	PAR3L_HUMAN	Partitioning defective 3 homolog B	133,097/8.54	72	11%	11	Cell cycle and cell division.
**1378**	GARL3_HUMAN	GTPase-activating Rap/Ran-GAP domain-like protein 3	113,808/7.57	60	9%	8	Regulation of small GTPase mediated signal transduction.

^1^ Spot number as given in 2-DE master gels; ^2^ accession number as in SwissProt database; ^3^ theoretical mass (MW) and Pi revealed in SwissProt database; ^4^ according to SwissProt database mascot score higher than 56 and *p* < 0.05 from Mascot search on proteomics data were considered; ^5^ Percentage of amino acids sequence coverage of matched peptides for the identified proteins; ^6^ Number of peptides matched by MALDI-TOF/MS for each identified protein.

**Table 2:** The String Network interaction of down-regulated cancer marker proteins in *N. nomurai* venom treated HepG2 cells along with KEGG pathways.

S.NO	Protein Name	String Interactions	KEGG Pathways
1	PCNA	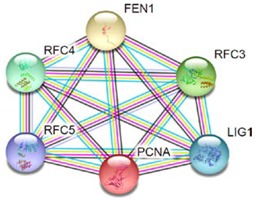	DNA replication, mismatch repair, nucleotide excision repair, and base excision repair.
2	HSPA5/GRP78	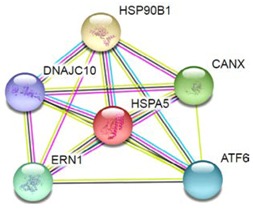	Protein processing in endoplasmic reticulum, thyroid hormone synthesis, antigen processing, and presentation.
3	G6P8	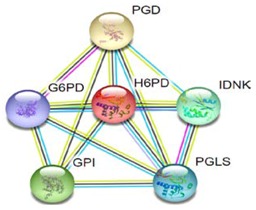	Microbial metabolism in diverse environments, carbon metabolism, glycolysis/gluconeogenesis, amino sugar and nucleotide sugar metabolism, starch and sucrose metabolism, pentose phosphate pathway, biosynthesis of amino acids, and pyruvate metabolism.	
4	EEF1G	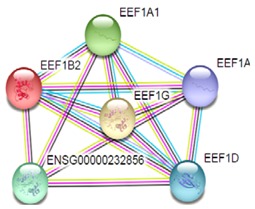	Ribosome Legionellosis and Legionellosis.
5	DNM1L	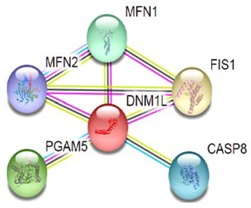	TNF signaling pathway.
6	NUSAP1	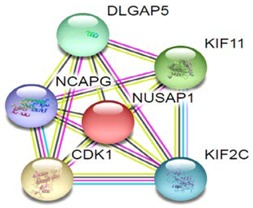	Mitotic sister chromatid, segregation, mitotic nuclear division, and cell division.
7	HSP90	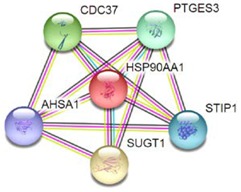	Protein processing in endoplasmic reticulum, antigen processing and presentation, NOD-like receptor signaling pathway, progesterone-mediated oocyte, prostate cancer, and estrogen signaling pathway.

## Supplementary Materials

The following are available online at http://www.mdpi.com/2072-6651/10/5/194/s1, Table S1: The proteins with declined relative abundance after NnV treatment, Table S2: The proteins with increased relative abundance after NnV treatment.
