# Supplementary material for: Proteomic Investigation to Identify Anticancer Targets of Nemopilema nomurai Jellyfish Venom in Human Hepatocarcinoma HepG2 Cells
Source: Toxins (Basel). 2018 May 10;10(5):194. doi: 10.3390/toxins10050194 (PMC5983250; doi:10.3390/toxins10050194)
Supplement: Supplementary file 1 [file toxins-10-00194-s001.pdf]

Supplementary Materials: Proteomic investigation to identify anticancer targets of *Nemopilema nomurai* jellyfish venom in human hepatocarcinoma HepG2 cell

Table S1. The proteins with declined relative abundance after NnV treatment. Proteins with ≥ 1.5 fold change and with statistically significance p value ≤ 0.05 were considered. Fold change and p value of individual candidate protein is mentioned.

Spot no	Accession no	Protein name	p value	Fold change	Relative spot intensity
2277	PCNA_HUMAN	Proliferating cell nuclear antigen	0.034	1.9	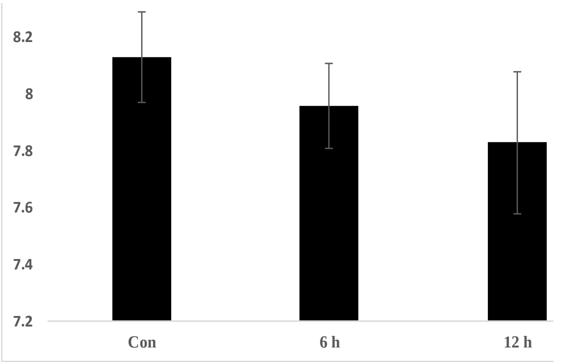
1648	KIF28_HUMAN	Kinesin like protein KIF 28P	0.037	3.7	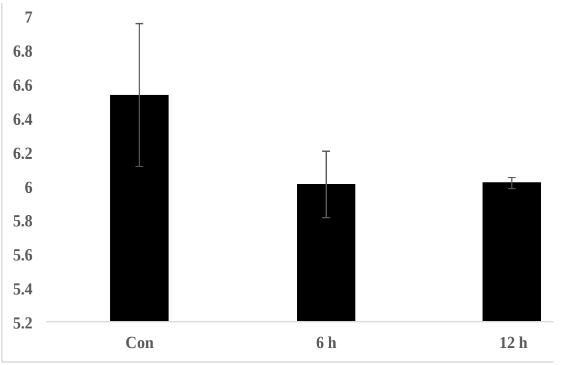
2299	GRP78_HUMAN	78 kDa glucose - regulated protein	0.032	3.3	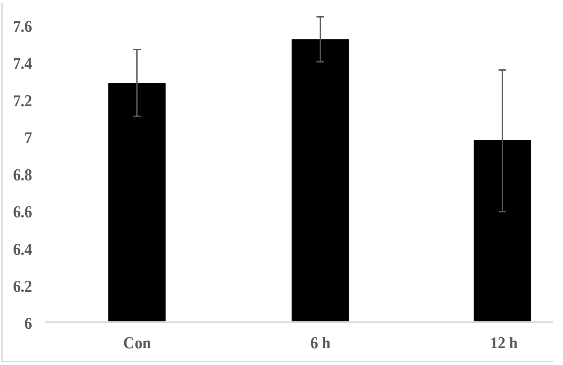

2008	AHSA1_HUMAN	Activator of 90kDa heat shock protein ATPase homolog 1	0.016	1.5	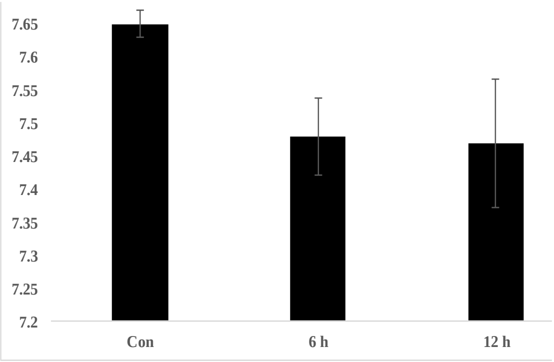
2245	NDUF7_HUMAN	NADH dehydrogenase [ubiquinone] complex1,assembly factor 7	0.029	2.9	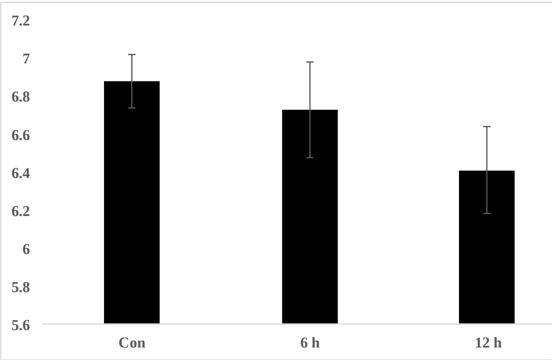
1768	NUSAP_HUMAN	Nucleolar and spindle-associated protein 1	0.024	1.8	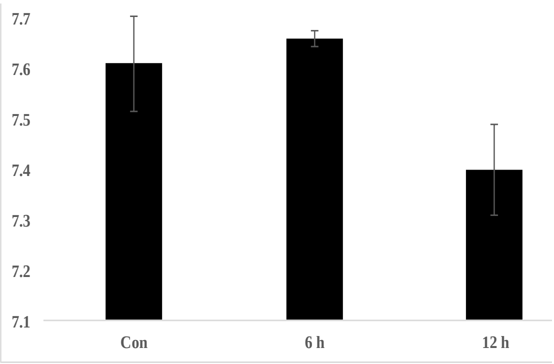
1978	DNM1L_HUMAN	Dynamin-1-like protein	0.015	2.2	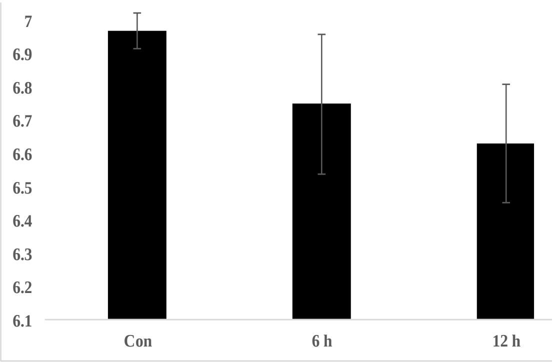

2183	ATLA3_HUMAN	Atlastin-3	0.029	6.8	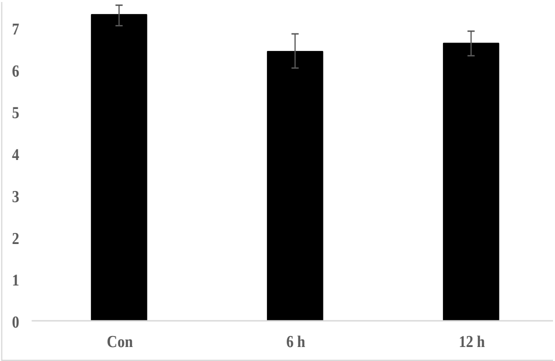
1772	HNRH1_HUMAN	Heterogeneous nuclear ribonucleo protein H	0.042	7.5	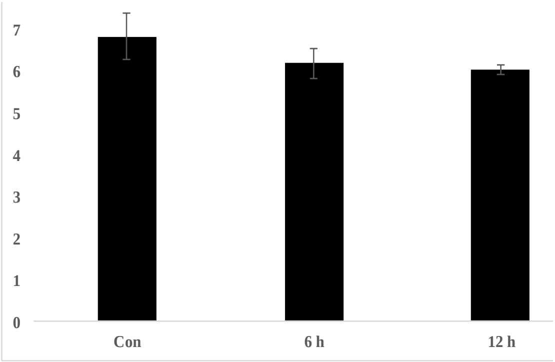
1817	EF1G_HUMAN	Elongation factor 1-gamma	0.041	3.8	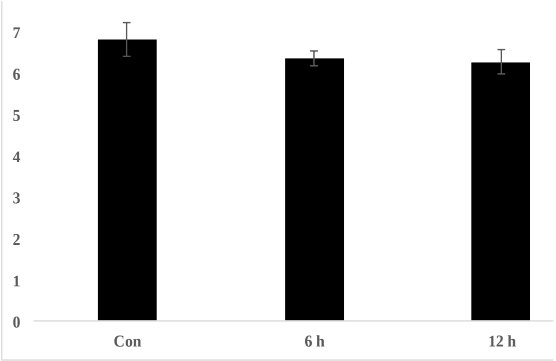
1665	G6PD_HUMAN	Glucose-6-phosphate 1-dehydrogenase	0.002	4.5	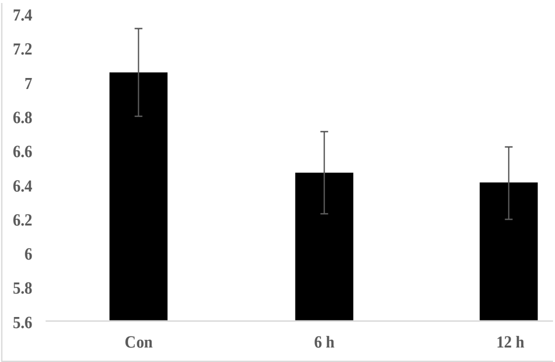

2245	ATP23_HUMAN	Mitochondrial innear membrane protease ATP23 homolog	0.028	2.9	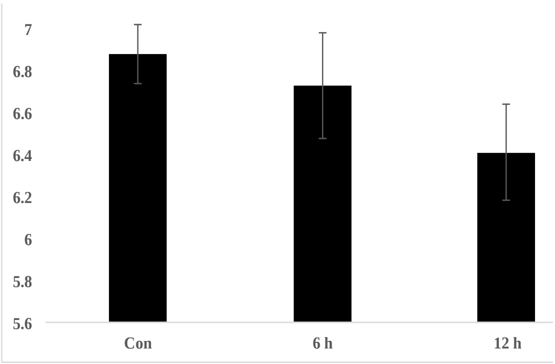
2089	DNM3A_HUMAN	DNA (Cytosine-5)-methyl transferase 3A	0.017	2.8	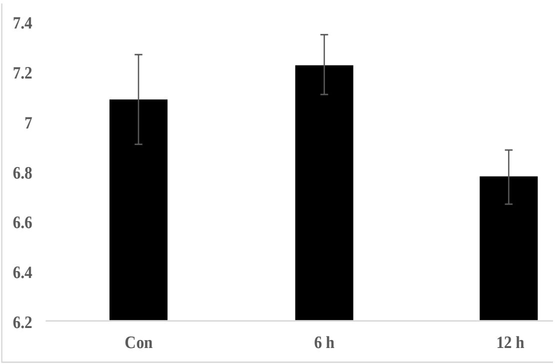
1648	TYW1_HUMAN	S-adenosyl-L-methionine dependent tRNA 4-demethyl wyosine synthase	0.037	3.7	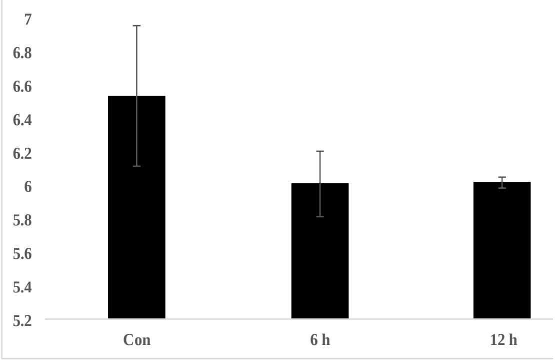

8

Table S2. The proteins with increased relative abundance after NnV treatment. Proteins with ≥ 1.5 fold change and with statistically significance p value ≤ 0.05 were considered. Fold change and p value of individual candidate protein is mentioned.

Spot no	Accession no	Protein name	p value	Fold change	Relative spot intensity
1646	CP21A_HUMAN	Steroid 21-hydroxylase	0.036	3.8	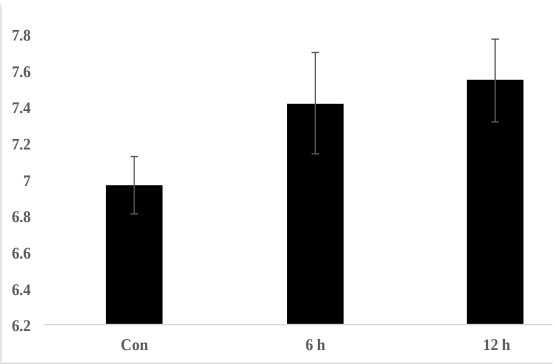
2353	AICDA_HUMAN	Single-stranded DNA cytosine deaminase	0.05	4.8	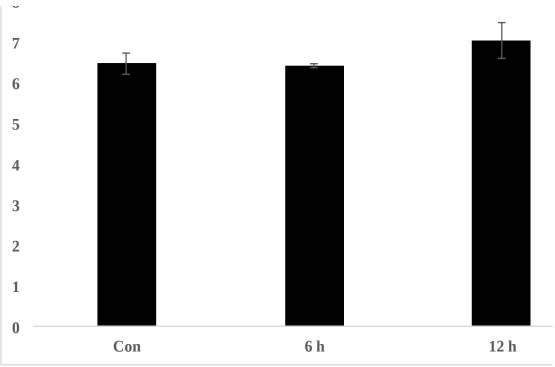
1906	UBP15_HUMAN	Ubiquitin carboxyl-terminal hydrolase 15	0.016	3.1	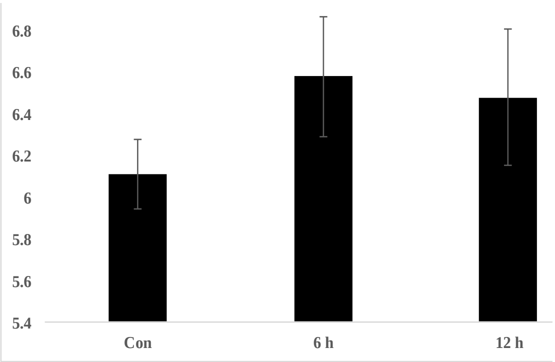

2029	PI4KA_HUMAN	Phosphatidylinositol 4-kinase alpha	0.035	3.4	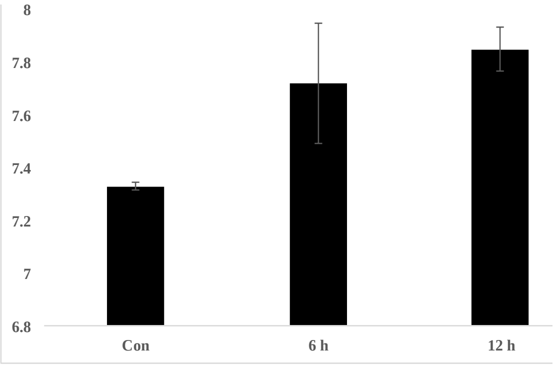
2014	SYHC_HUMAN	Histidine -t RNA Ligase	0.003	1.9	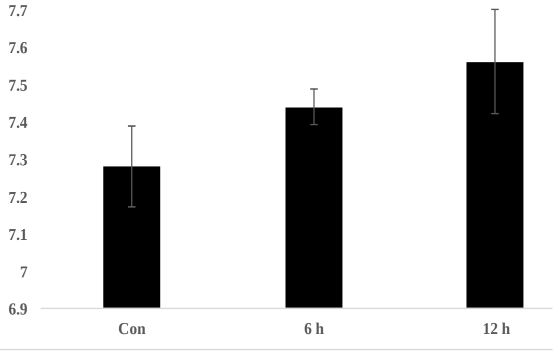
821	DHB13_HUMAN	17-beta-hydroxysteroid dehydrogenase 13	0.017	1.6	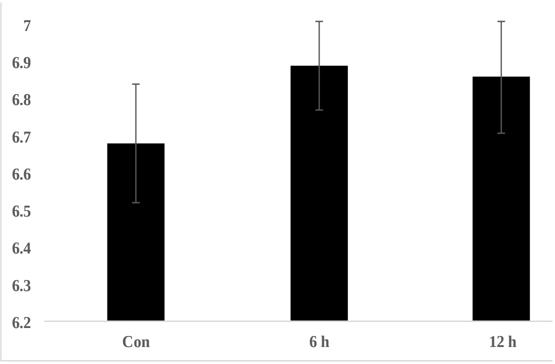
2841	SARM1_HUMAN	Sterile alpha and TIR motif- containing protein 1	0.02	3	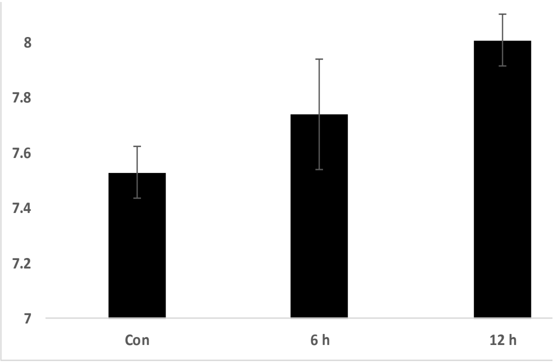

2282	ZSC31_HUMAN	Zinc finger and SCAN domain-containing protein 31	0.023	3.1	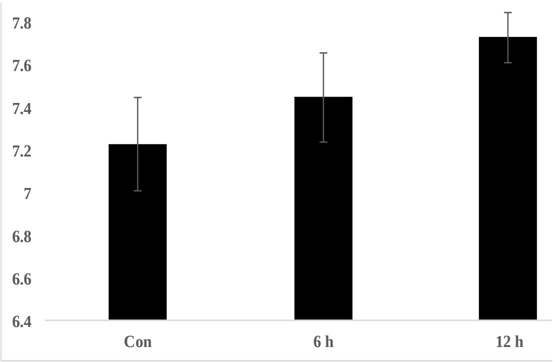
2217	PAR3L_HUMAN	Partitioning defective 3 homolog B	0.047	3.3	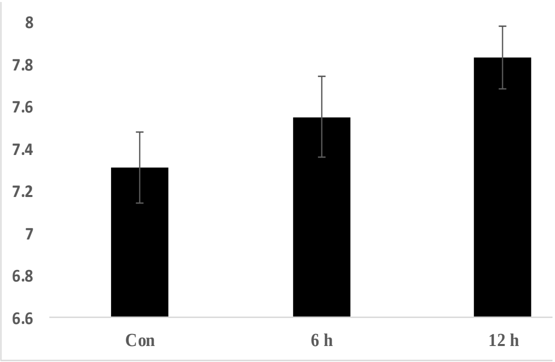
1378	GARL3_HUMAN	GTPase-activating Rap/Ran-GAP domain-like protein 3	0.019	3.3	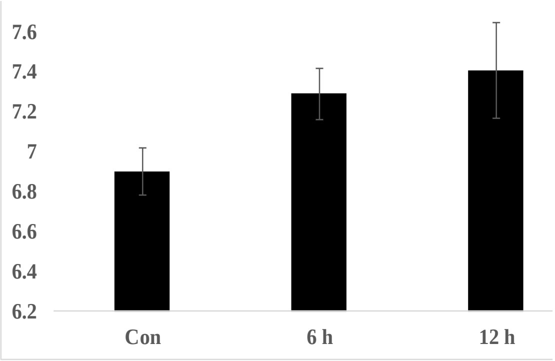
